# Characterization of the endogenous retrovirus insertion in *CYP19A1* associated with henny feathering in chicken

**DOI:** 10.1186/s13100-019-0181-4

**Published:** 2019-08-28

**Authors:** Jingyi Li, Brian W. Davis, Patric Jern, Ben. J. Dorshorst, Paul B. Siegel, Leif Andersson

**Affiliations:** 1 Department of Veterinary Integrative Biosciences, College of Veterinary Medicine and Biomedical Sciences, Texas A&M University, College Station, TX 77843 USA; 2 Science for Life Laboratory, Department of Medical Biochemistry and Microbiology, Uppsala University, SE-751 23 Uppsala, Sweden; 3 Department of Animal Breeding and Genetics, Swedish University of Agricultural Sciences, SE-7507 Uppsala, Sweden; 4 Department of Animal and Poultry Sciences, Virginia Polytechnic Institute and State University, Blacksburg, Virginia, 24061 USA

**Keywords:** Chicken, Henny feather, Aromatase, Endogenous retrovirus, ERV, LTR

## Abstract

**Background:**

Henny feathering in chickens is determined by a dominant mutation that transforms male-specific plumage to female-like plumage. Previous studies indicated that this phenotype is caused by ectopic expression in skin of *CYP19A1* encoding aromatase that converts androgens to estrogen and thereby inhibits the development of male-specific plumage. A long terminal repeat (LTR) from an uncharacterized endogenous retrovirus (ERV) insertion was found in an isoform of the *CYP19A1* transcript from henny feathering chicken. However, the complete sequence and the genomic position of the insertion were not determined.

**Results:**

We used publicly available whole genome sequence data to determine the flanking sequences of the ERV, and then PCR amplified the entire insertion and sequenced it using Nanopore long reads and Sanger sequencing. The 7524 bp insertion contains an intact endogenous retrovirus that was not found in chickens representing 31 different breeds not showing henny feathering or in samples of the ancestral red junglefowl. The sequence shows over 99% sequence identity to the avian leukosis virus ev-1 and ev-21 strains, suggesting a recent integration. The ERV 3’LTR, containing a powerful transcriptional enhancer and core promoter with TATA box together with binding sites for EFIII and Ig/EBP inside the *CYP19A1* 5′ untranslated region, was detected partially in an aromatase transcript, which present a plausible explanation for ectopic expression of aromatase in non-ovarian tissues underlying the henny feathering phenotype.

**Conclusions:**

We demonstrate that the henny feathering allele harbors an insertion of an intact avian leukosis virus at the 5’end of *CYP19A1*. The presence of this ERV showed complete concordance with the henny feathering phenotype both within a pedigree segregating for this phenotype and across breeds.

**Electronic supplementary material:**

The online version of this article (10.1186/s13100-019-0181-4) contains supplementary material, which is available to authorized users.

## Background

**Fig. 1:**
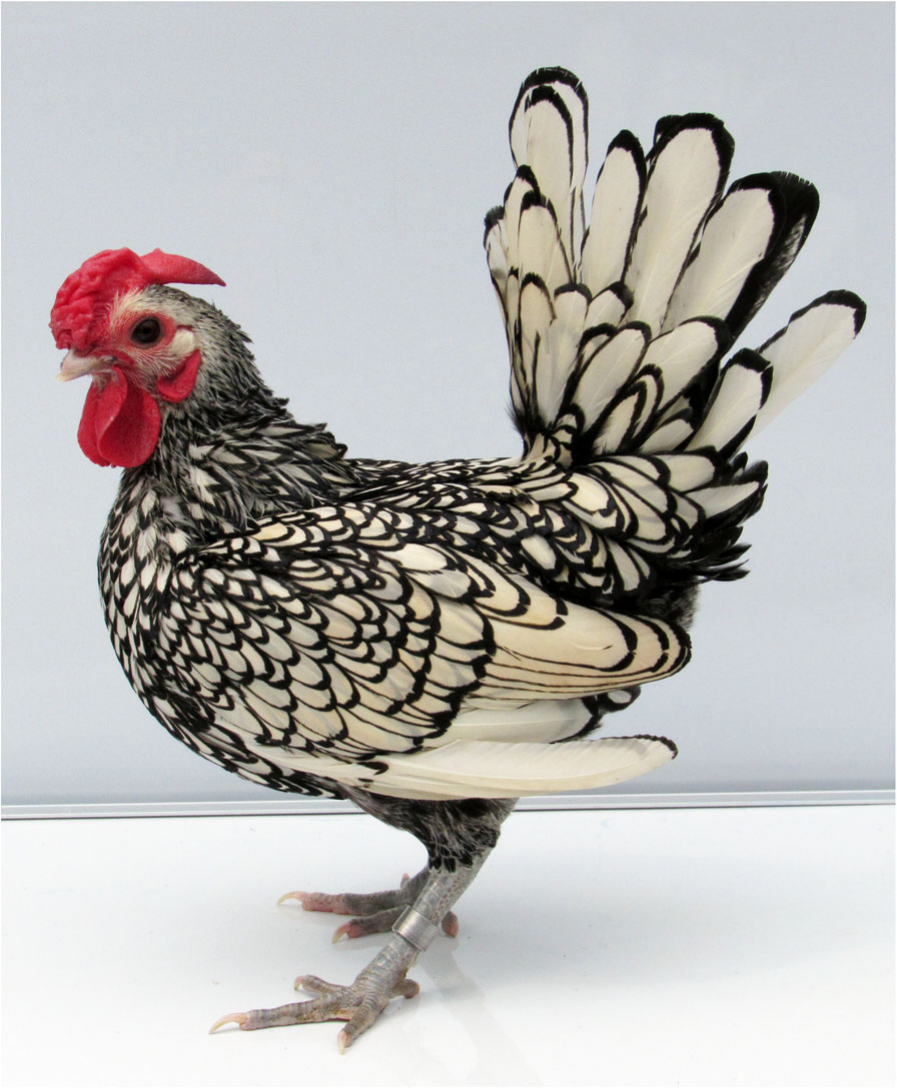
Adult male silver Sebright Bantam chicken expressing the henny feathering phenotype. (Photo: Jingyi Li)

Plumage color is a striking and variable aspect of sexual dimorphism in avian species. Males often exhibit a showy plumage during mating season, which is an example of a compromise between sexual selection and avoiding predation by camouflage color outside the breeding season [1, 2]. Understanding the molecular mechanisms of male feathering therefore involves basic principles in developmental and evolutionary biology. The wild ancestor of the domestic chicken, the red junglefowl, shows a spectacular sexual dimorphism like many other pheasant species. However, some domestic chickens carry the dominant henny feathering allele that makes the plumage of males resemble females [3]. This phenotype offers a model to study the molecular basis for sexual dimorphism in avian species and has therefore been extensively studied [4]. The henny feathering trait was first observed by Sir John Sebright in 1800 [5], and became a distinguishing feature of the Sebright Bantam breed (Fig. 1). Other henny feathered chicken breeds such as the Golden Campine likely received this trait from Sebright due to interbreeding by chicken fanciers [6]. The henny feathering (*Hf*) mutation is desirable when breeding fancy chickens because it allows males to show intrafeather patterns which are normally interrupted in males homozygous for the wild-type allele (*hf*^*+*^). In many other show chicken breeds, only females can meet the criteria for exhibition because of their uniformity of intrafeather patterns. However, in Sebright and Golden Campine, males are preferred because *Hf* allows them to express uniform intrafeather patterning while retaining other male characters, like larger combs and elongated tail feathers [7].

The plumage in *hf*^*+*^ male chickens has a shiny appearance with pointy tips in saddle and hackle feathers. Hens have a more ‘dun’ plumage with duller, rounder feathers. Testis removal does not affect the appearance of these feathers, however ovary removal in females induces a male-shiny phenotype [8]. The typical feathering of hens is produced by the action of estrogen, predominantly produced in the ovaries through aromatase conversion from androgens [9]. In early studies of the henny feathering phenotype, castration of gonads [10, 11], castration followed by treatment with different androgens [12, 13], as well as transplantation of gonads [14] and skin [15] suggested that the changes induced by *Hf* must reside in the skin, decreasing testosterone and increasing estrogen, resulting in a feminized plumage [13]. This is consistent with current knowledge that *Hf* is caused by ectopic expression of aromatase in the skin of Sebright and Campine chickens [4, 16–21]. In adult wild-type chickens, aromatase is detectable only in ovaries and hypothalamus [17]. In males carrying the dominant *Hf* allele, such conversion also happens in non-ovarian tissues such as skin [4]. *Hf* is co-dominant with respect to aromatase activity, as heterozygotes have an enzyme activity intermediate between the two homozygotes. It is dominant with respect to feathering, as a single dose of the *Hf* allele leads to the production of adequate estrogen in skin to induce henny feathering [18]. Furthermore, a study on transgenic cocks overexpressing aromatase also resulted in female feathering resembling the *Hf* phenotype [22]. In other studies, after the injection of an aromatase inhibitor during embryonic stage, female chickens developed testes and had the behavior and physical appearance of males [9, 23].

A long terminal repeat (LTR) from an uncharacterized inherited endogenous retrovirus (ERV) was found in the cDNA of *CYP19A1*, which encodes aromatase in chicken. This LTR was hypothesized to act as a promoter inducing ubiquitous upregulation of *CYP19A1* and thus inducing aromatase activity in non-ovarian tissues [21]. However, the ERV was not characterized in detail, and the mechanism causing ectopic aromatase expression is not fully understood. Here we used publicly available whole genome sequence (WGS) data from chickens with and without henny feathering to identify the flanking sequence of the insertion, and long-read Nanopore as well as Sanger sequencing to characterize the locus. We show that the insertion is indeed an intact 7524 bp recently integrated ERV, which is related to avian leukosis viruses and missing in the ancestral red junglefowl as well as in related chicken breeds.

## Results

### Insertion site identified using WGS data

**Fig. 2:**
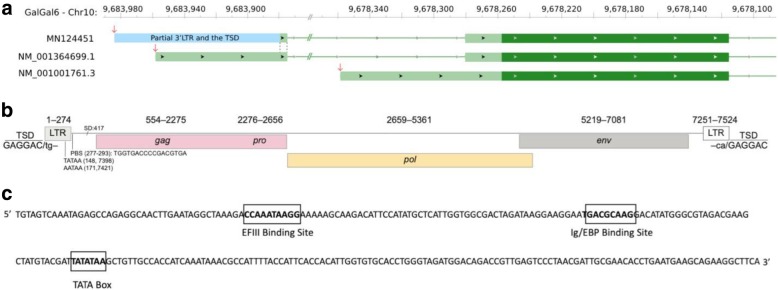
Structure of the ERV insertion associated with henny feathering (Hf_ERV). **a**) Schematic drawing of different *CYP19A1* transcripts. Transcription start sites are indicated with red arrows. The top row is the structure of Hf_ERV induced transcription, identified by 5’RACE and submitted to GenBank (accession number MN124451). Middle row is the long transcript isoform present in wild-type chicken. The bottom row is the short wild-type isoform. Coding sequences, UTRs, and introns positions are based on NCBI and are indicated by dark green boxes, light green boxes, and light green lines, respectively; black arrows mark the orientation of transcription. Dash lines represents the shared 4 bp sequence of exon 1 between these 2 transcripts, other exons are identical. **b**) Positions and sizes of the two flanking LTRs and the internal retroviral *gag*, *pro*, *pol*, and *env* genes. TSD = target site duplication; PBS = primer binding site; SD = putative splice donor motif. **c**) Sequence and annotation of the two identical LTRs showing binding sites for EFIII and Ig/EBP, as well as a TATA box promoter

Guided by the localization of the putative ERV adjacent to *CYP19A1* [21], we used publicly available whole genome sequence data from individually sequenced chickens and from pooled sequencing in total representing 32 breeds of domestic chicken, and in addition five individually sequenced and two pools of red junglefowl (Additional file 1: Table S1). We focused the analysis on the 200 kb region surrounding *CYP19A1* to identify structural variants present in Sebright chickens (*Hf/−*) and not present in chickens from other breeds (*hf*^*+*^*/hf*^*+*^). Only one structural variant met this criterion, and was present at position 9,683,879 bp on chromosome 10 of GalGal6, which is in the 5′ untranslated region (5’UTR) of *CYP19A1* (Fig. 2a). The sequence of soft-clipped reads surrounding the insertion position confirmed the presence of an LTR (Additional file 5: Figure S1).

### Sequencing of the *Hf* associated ERV insertion

**Fig. 3:**
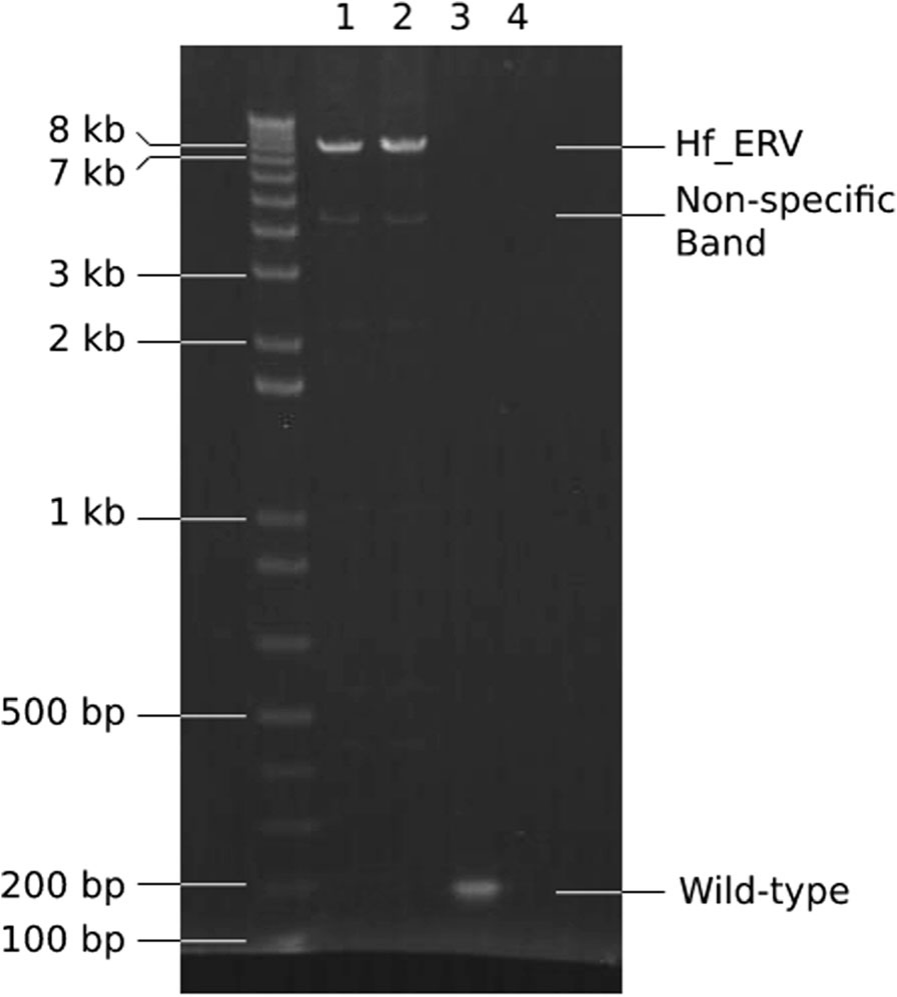
PCR amplicons with or without the entire Hf_ERV insertion. All amplicons were amplified by the same pair of primers, which is flanking the Hf_ERV insertion site. A 7.5 kb amplicon suggests the presence of Hf_ERV, while a 163 bp amplicon suggests at least one copy of the wild-type chromosome which is lacking Hf_ERV. Lane 1 and 2 are two Sebright individuals with Hf_ERV. Lane 3 is a red junglefowl individual which is wild-type. Lane 4 is no template control for PCR

PCR amplification of the *Hf* insertion produced an amplicon of about 7.5 kb from all *Hf/−* chickens whereas only short amplicons (163 bp) were obtained from wild-type chickens (Fig. 3). The polished de novo contig from long-read sequencing (GenBank accession number MK937054) was parsed for retroviral similarity, revealing high sequence identity to avian leukosis virus. A sequence comparison of the henny feathering associated ERV (Hf_ERV), described in this study, with 140 published avian leukosis viruses identified 50 retroviruses with over 90% identity (Additional file 2: Table S2). The highest sequence identity (99.4%) was obtained against the ev-21 strain, which shares an origin with the Rous sarcoma virus (Additional file 6: Figure S2). Examination of the Hf_ERV sequence motifs revealed intact *gag*, *pro*, *pol* and *env* genes flanked by two identical 274 bp LTRs (Fig. 2b). Each LTR contains a TATA box promoter sequence [24], as well as binding sites for the avian serum response factor EFIII and Ig/EBP [25] (Fig. 2c), a ubiquitously expressed immunoglobulin enhancer binding protein. Immediately flanking the LTRs, we found identical 6 bp sequences, GAGGAC, identified as the chromosomal target site duplications (TSD) formed during integration (Fig. 2b).

### 5’RACE of *CYP19A1* reveals start of transcription in Hf_ERV

5’RACE experiments using *CYP19A1*-specific primers (Additional file 3: Table S3) and mRNA isolated from the skin of a one-day-old Sebright chick (genotyped as *Hf*/*Hf*) revealed only one transcript isoform (GenBank accession number MN124451). The sequence data showed that the transcription start is 24 bp downstream of the 3’LTR TATA box. It includes the remaining 99 bp of the 3’LTR together with the TSD, as well as partial 5’UTR (4 bp) of the wild-type *CYP19A1* transcript (NM_001364699.1), and the *CYP19A1* coding sequence (CDS) (Fig. 2a). These sequence features suggest that the 3’LTR TATA box of Hf_ERV acts as the promoter that induce the transcription of the Hf_ERV and *CYP19A1* fusion transcript, which uses the same splicing sites as the wild-type transcript. The cDNA sequence was fully consistent with the genomic sequence of the Hf_ERV insertion.

### Hf_ERV-induced transcripts are detected in Sebright but not in wild-type chicken

**Fig. 4:**
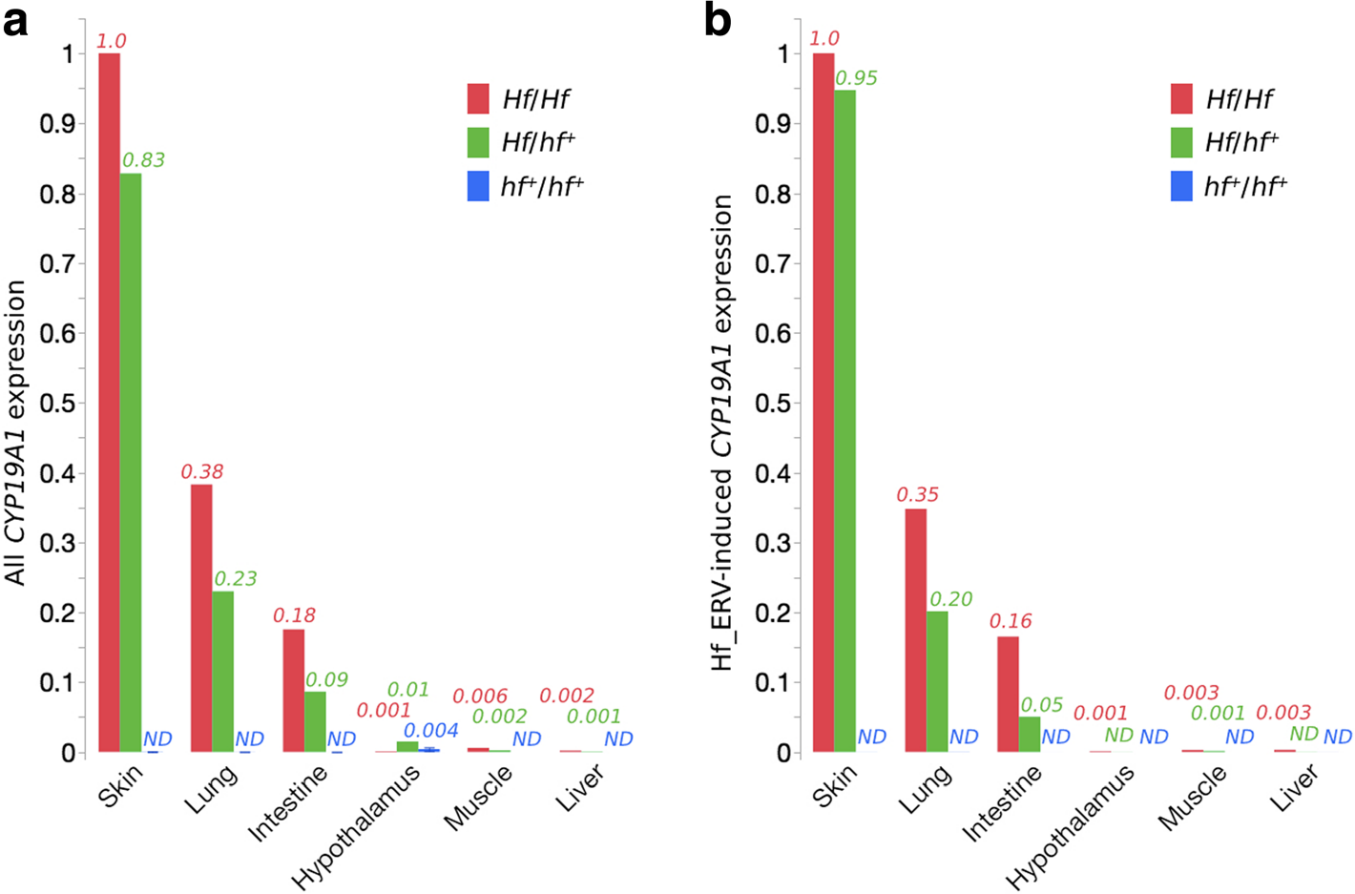
RT-qPCR analysis of *CYP19A1* coding region (All *CYP19A1* expression) and Hf_ERV-induced *CYP19A1* expression. Relative expressions are reported as proportion to the expression level in the skin of Sebright, and are labeled as italic numbers on the top of each bar. ND means not detected. *CYP19A1* expression was normalized against *GAPDH* expression. Sample size: *Hf*/*Hf* (*N* = 1), *Hf*/*hf*^*+*^ (N = 1), *hf*^*+*^/*hf*^*+*^ (*N* = 2). **a**) *CYP19A1* coding region is detected as the sum of all known transcripts, including Hf_ERV-induced expression. **b**) Hf_ERV-induced expression detected using Hf-ERV forward primer

We explored the *CYP19A1* expression pattern across six tissues (skin, lung, intestine, hypothalamus, muscle, and liver) and using two sets of primer combinations, one encompassing only coding sequences and expected to amplify all *CYP19A1* transcripts and the other only detecting HF_ERV-induced transcripts (Fig. 4). In wild-type (*hf*^*+*^/*hf*^*+*^) one-day-old chicks, the expression of the *CYP19A1* coding region was only detected in hypothalamus (Fig. 4a). For Sebright chicks a very similar expression pattern was observed with the two sets of primers, with high ectopic expression in skin, lung, and intestine, and low expression in hypothalamus, muscle, and liver (Fig. 4a, b). The results strongly suggest that the Hf_ERV-induced transcript, starting at the 3’LTR TATA box, is the only major isoform responsible for ectopic expression of *CYP19A1* in *Hf* chicks. The level of ectopic expression was higher in the homozygote (*Hf*/*Hf*) than in the heterozygote (*Hf*/*hf*^*+*^) as expected.

### Segregation of the henny feathering phenotype in a backcross population

**Fig. 5:**
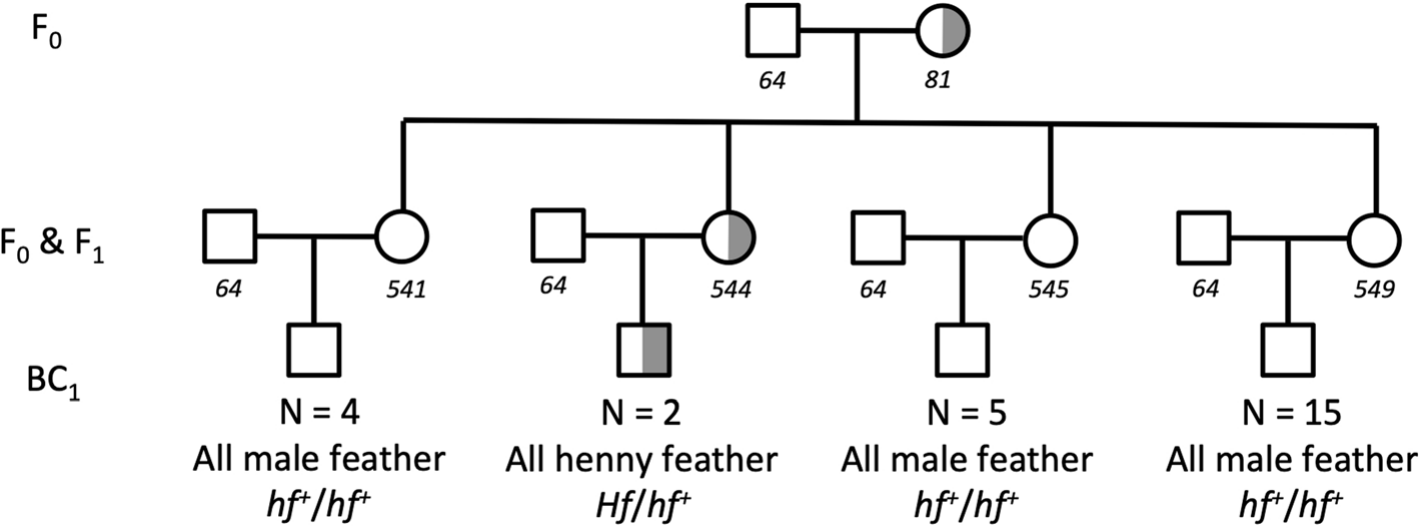
A portion of the backcross population pedigree. Animal IDs are indicated with italic numbers. Open glyphs indicate that the individual does not carry the Hf_ERV insertion, while half shaded indicates heterozygosity. ID 64 is a Hamburg wild-type male (*hf*^*+*^/*hf*^*+*^). He was used to generate both F_1_ and backcross progeny. The *Hf* genotype of females cannot be inferred by their phenotype because henny feathering is a male-limited trait. But their genotype can be inferred based on pedigree data. Therefore, female 544 and 81 are *Hf*/*hf*^*+*^ and all other females should be *hf*^*+*^/*hf*^*+*^

To further explore the genotype-phenotype association for henny feathering we analyzed segregation data in a backcross pedigree. Four F_0_ Sebright females homozygous for Hf_ERV (*Hf*/*Hf*) were mated with three Silver Spangled Hamburg males (*hf* ^+^/*hf* ^+^), which produced 17 F_1_ females. These were backcrossed to F_0_ males to produce 60 backcross males. The henny feathering phenotype was observed in 27 progenies, while 33 were wild-type, consistent with an expected 1:1 segregation (*P* = 0.44). A fifth F_0_ Sebright female was heterozygous for Hf_ERV and produced 4 F_1_ females, one (ID: 544) was heterozygous (*Hf*/*hf*^+^) while the other three (ID: 541, 545, 549) were homozygous wild-type (Fig. 5). The segregation of the henny feathering phenotype among their back-cross males was in perfect agreement with the genotype determined based on the Hf_ERV insertion. The result from this pedigree is fully consistent with the dominant inheritance of henny feathering and shows that *Hf* is not fixed in this Sebright population. We estimated the allele frequency of *Hf* to 0.80 in a sample of Sebright chickens (*n* = 20). One of the two Campine chickens that we genotyped was homozygous mutant (*Hf*/*Hf*) while the other was homozygous wild-type (*hf*^+^/*hf*^+^, Additional file 4: Table S4), indicating that *Hf* is not fixed in this breed either. None of these samples had phenotypic information because they were females or this specific phenotype was not recorded.

## Discussion

*Hf* was previously mapped to the linkage group E29C09W09 (cited by Carefoot [26]), which is now known to reside on chicken chromosome 10. Previous studies indicated that an LTR from a, putative ERV upstream of *CYP19A1* might cause henny feathering by acting as a *cis*-regulatory mutation driving ectopic expression of aromatase [21, 27]. In our study, a 7524 bp ERV within the 5’UTR of *CYP19A1* was completely associated with *Hf*. The genomic sequence documented in our study matches 83 bp of the cDNA sequence reported by Matsumine et al. [21] whereas 45 bp of the cDNA shows multiple sequence differences (Additional file 7: Figure S3), possibly due to sequencing errors in the previous sequence.

Our study demonstrates that ERVs in the chicken genome can have profound phenotypic effects. Other examples include a retroviral insertion in the tyrosinase gene causing the recessive white plumage color [28] and an EAV-HP insertion in the promoter region of *SLCO1B3,* which upregulates gene expression causing the blue eggshell phenotype [29, 30]. We found two identical 274 bp LTRs in the Hf_ERV, showing high sequence identity to avian leukosis virus strain ev-21, each containing a powerful transcriptional enhancer and core promoter with a TATA box (Fig. 2c). The 5’RACE experiment strongly suggested that the proximity of the promoter in the 3’LTR, and its binding sites for EFIII and Ig/EBP, to *CYP19A1* (Fig. 2) are the drivers of ectopic expression of aromatase and thereby the conversion of androgen to estrogen in the skin and other non-ovarian tissues. Given the identical LTRs, it is conceivable that expression of ERV genes could be induced from the 5’LTR. Hf_ERV has intact *gag*, *pro*, *pol*, and *env* genes, as well as intact TSDs immediately flanking the two identical LTRs, indicating a recent insertion (Fig. 2b). The *Hf* insertion most likely occurred in one of the close ancestors of the “hen-tailed Bantam cock” found by Sir John Sebright in 1800 [5]. It also suggests that the Hf_ERV could still be active and potentially replicate further. We observed that the *Hf* allele was not fixed neither in the Sebright nor in the Campine. This is probably due to reduced fertility of males that are homozygous for *Hf* [17, 31]. Male heterozygotes produce more offspring, which thus reduces selection against the *hf*^*+*^ allele. The reduced male fertility is likely caused by the ectopic expression of aromatase in testis [17, 32], or possibly expression of Hf_ERV which could reduce fertility and hatchability of the host chicken [33]. Therefore, functional studies are needed to determine the direct effects of the Hf_ERV insertion.

Two isoforms of *CYP19A1* transcripts have been documented in wild-type chickens, NM_001364699.1 and NM_001001761.3. Their coding regions are identical, however NM_001364699.1 is longer because of an extended 5’UTR (Fig. 2a). Although both transcripts are expressed in cerebrum, only the shorter transcript is detected in ovary [34], which is consistent with previous reports that aromatase is detectable only in ovary and hypothalamus in wild-type chickens [17]. In both *Hf*/*−* and *hf*^+^/*hf*^+^ chickens, the shorter (NM_001001761.3) isoform can be detected in the ovary but not in fibroblasts [21]. However, because a transcript (MN124451) corresponding to NM_001364699.1, with an extended UTR and partial LTR sequence (Fig. 2a), can be detected in both ovary and fibroblasts from *Hf*/*−* chickens [21], it is most likely responsible for the aromatase activity in all extragonadal tissues. This is supported by our qPCR result showing that Hf_ERV-induced transcripts are detected at high levels in skin, lung, and intestine, but also at low level in liver, muscle, and hypothalamus in Sebright chickens. The observed *CYP19A1* expression pattern in Sebright chicks (skin > lung > intestine > > muscle > liver; Fig. 4) matches previous reports about estrogen formation [17] and aromatase activity [35] in Sebright chickens. We propose that the effect of the *Hf* mutation is changing the expression pattern of the long isoform, represented by NM_001364699.1, from hypothalamus-specific to more broadly expressed and possibly without affecting the ovary-specific transcript.

## Conclusions

This study shows that the henny feathering trait in chicken is associated with the insertion of an intact avian leukosis virus in the 5’UTR of *CYP19A1*. The insertion of strong viral promotors in this region appears as a plausible causal mutation for the ectopic expression of *CYP19A1* underlying henny feathering.

## Methods

### Animals

A trait mapping population was initiated from 8 chickens purchased from Murray McMurray Hatchery (www.mcmurrayhatchery.com, Webster City, Iowa, USA) to examine the segregation of *Hf*. They consisted of 3 Silver Spangled Hamburg males and 5 Silver Sebright females crossed to generate the F_1_ generation. Matings between 21 F_1_ females and 3 F_0_ Hamburg males produced 86 backcross males. Photos for phenotyping the backcrosses were taken (focusing on the hackle and saddle feathers which show the most distinct sexual dimorphism) at 12 weeks. The putative ERV insertion associated with *Hf* was characterized using 18 DNA samples, 16 Sebright and 2 Campine chickens, and 17 of these were sequenced using Nanopore long reads (Additional file 4: Table S4).

Liver tissues for DNA preparation and tissue samples (liver, lung, hypothalamus, muscle, intestine, and dorsal skin) for RNA isolation were collected from two one-day-old Silver Sebright chicks purchased from Ideal Poultry (http://www.idealpoultry.com/, Cameron, Texas, USA). The same tissues for RNA samples were collected from two one-day-old red junglefowls from a colony of red junglefowl kept in Texas A&M University’s Poultry Research Center.

### Whole genome sequence (WGS) analyses

Illumina paired-end FASTQ data for 82 individuals or pooled samples from public databases, including one Sebright pool (Additional file 1: Table S1), aligned to the red junglefowl genome assembly version GalGal6 using BWA, sorted with SAMtools, and variants were called with GATK HaplotypeCaller 3.8 according to Broad best practices [36]. Structural variants were called with Lumpy in single-sample mode [37].

### Genotyping

Individual DNA samples were isolated from blood or liver using Qiagen, Puregene Tissue Core Kit B, DNA Isolation Protocol for Avian Blood with minor modifications. Each of the backcrossed males, F_1_ females, F_0_ and 18 chickens from Sebright or Campine lines (Additional file 4: Table S4) were genotyped by PCR (standard protocol for TAKARA PrimeSTAR GXL DNA Polymerase) with the forward primer HFEV_F and reverse primer HFEV_R2 (Additional file 3: Table S3).

### Amplicon sequencing and assembly

Purified amplicons, encompassing the entire *Hf* insertion from 17 chickens, were barcoded using the Oxford Nanopore (ONT) Rapid Barcoding Kit (#SQK-RBK004) and sequenced on a single R9.5.1 flowcell. Raw nanopore FAST5 reads were converted to FASTQ using Albacore v2.3.4 (ONT), and assembled de novo using Canu 1.8 [38]. An 85% majority consensus of the *Hf* insertion was derived from all 17 individuals, and the amplicon from one individual was manually polished by Sanger sequencing. Sanger primers are listed in Additional file 3: Table S3. PCR products for Sanger sequencing were generated via two-step nested PCR, which used purified amplicon from the genotyping PCR as the template to avoid amplification of homologous regions in the chicken genome. The polished sequence was used in BLAST searches to identify the most similar sequences, avian leukosis viruses. The consensus was aligned with 140 published avian leukosis viruses (Additional file 2: Table S2), and a maximum likelihood phylogeny was generated using RAxML 8.2.12 using the GTR + gamma nucleotide substitution model with 100 bootstraps [39]. Annotation of the sequence was performed with RepeatMasker for LTRs and the EBI-EMBL Pfam database for retroviral protein coding sequences. Retroviral sequence features were characterized by RetroTector [40].

### 5′ rapid amplification of cDNA ends (5’RACE)

To determine the 5′ ends of the ectopically expressed *CYP19A1* transcript in the skin of henny feathering chicken, 5’RACE experiments were performed with the kit Rapid Amplification of cDNA Ends (Invitrogen), according to the manufacturer’s protocol. The *CYP19A1* gene-specific primers were designed for cDNA synthesis (Hf_RACE_R1, Additional file 3: Table S3) and for subsequent PCR reactions and Sanger sequencing (Hf_RACE_R2, Additional file 3: Table S3).

### Quantitative real-time RT-PCR

Total RNA was extracted using Quick-RNA Miniprep Plus Kit (Zymo Research). First-strand cDNA was synthesized using SuperScript™ IV VILO™ Master Mix (Invitrogen). qPCRs were conducted with PowerUp™ SYBR™ Green Master Mix (Applied Biosystems) according to the manufacturer’s protocol. The products detected with the Roche LightCycler®480 using the standard protocol. Sequences of primers for the Hf_ERV induced transcript (Hf_qF and Hf_qR), the coding region of *CYP19A1* (CYP_qF and CYP_qR) and for house-keeping gene (GAPDH_qF and GAPDH_qR) are listed in Additional file 3: Table S3. Each PCR reaction has three technical replicates.

## Additional files

Additional file 1: **Table S1.** Public whole genome sequence data used in this study. (XLSX 11 kb)

Additional file 2: **Table S2.** Sequence comparison of the henny feathering endogenous retrovirus sequence (Hf_ERV) with 140 published avian leukosis viruses. (XLSX 16 kb)

Additional file 3: **Table S3.** PCR primers used in this study. (XLSX 10 kb)

Additional file 4: **Table S4.** List of DNA samples. (XLSX 9 kb)

Additional file 5: **Figure S1**. Aligned reads showing the insertion site of Hf_ERV on chicken chromosome 10. Aligned reads are shown in gray; multicolored “rainbow” reads are soft-clipped. Left clip and right clip sequences match the 5′ and 3′ of the LTR sequence, respectively. Discordant reads are in cyan which are well aligned in this figure (Chr10), while their mate pairs are aligned to Chr1, either to LOC770705 or LOC107052718, both retrovirus related genes, because there is no retrovirus sequence at this target site on chromosome 10 in the reference genome. Altogether, this alignment suggests a retrovirus insertion located between the left and right clips, which contains the 6 bp target site duplication (TSD) sequence. Reads are obtained from BioSample Accession SAMEA104432213 (Pool of Silver Sebright Bantam) and aligned to GalGal6. (DOCX 214 kb)

Additional file 6: **Figure S2.** Maximum likelihood phylogeny for the endogenous retrovirus associated with henny feathering (Hf_ERV). Hf_ERV and the 50 other retrovirus sequences with above 90% sequence identity to Hf_ERV. Bootstrap values are reported as percentages. Grey box indicates the clade containing Hf_ERV, ev-1 and ev21. (DOCX 475 kb)

Additional file 7: **Figure S3.** Comparison of sequences reported in this and previous studies. Alignment between a previously reported partial cDNA sequence of *CYP19A1* in Sebright chickens [21] and the retrovirus insertion detected in the present study (LTR). The base pair positions of the LTR sequence correspond to the complete LTR (5′ to 3′), which is 274 bp in length. (DOCX 140 kb)

## Data Availability

The annotated consensus sequence for Hf_ERV has been deposited in GenBank under the accession number MK937054. The 5′ partial sequence for Hf_ERV-induced *CYP19A1* transcripts, based on 5’RACE, has been deposited in GenBank under the accession number MN124451. The accession numbers of the 140 published avian leukosis virus sequences used in this article are included within Additional file 2: Table S2.

## Abbreviations

CDS: Coding sequence
*CYP19A1*: Cytochrome P450 family 19 subfamily A member 1
EFIII: Enhancer factor III
*Env*: Envelope
ERV: Endogenous retrovirus
Ev: Endogenous virus
*Gag*: Group specific antigen
Hf: Henny feather
Ig/EBP: Immunoglobulin/enhancer binding protein
LTR: Long terminal repeat
ONT: Oxford Nanopore
*Pol*: Polymerase
*Pro*: Protease
RACE: Rapid amplification of cDNA ends
TSD: Target site duplications
UTR: Untranslated region
WGS: Whole genome sequence
