# Supplementary figures and images for: Characterization of the endogenous retrovirus insertion in CYP19A1 associated with henny feathering in chicken
Source: Mob DNA. 2019 Aug 28;10:38. doi: 10.1186/s13100-019-0181-4 (PMC6712707; doi:10.1186/s13100-019-0181-4)

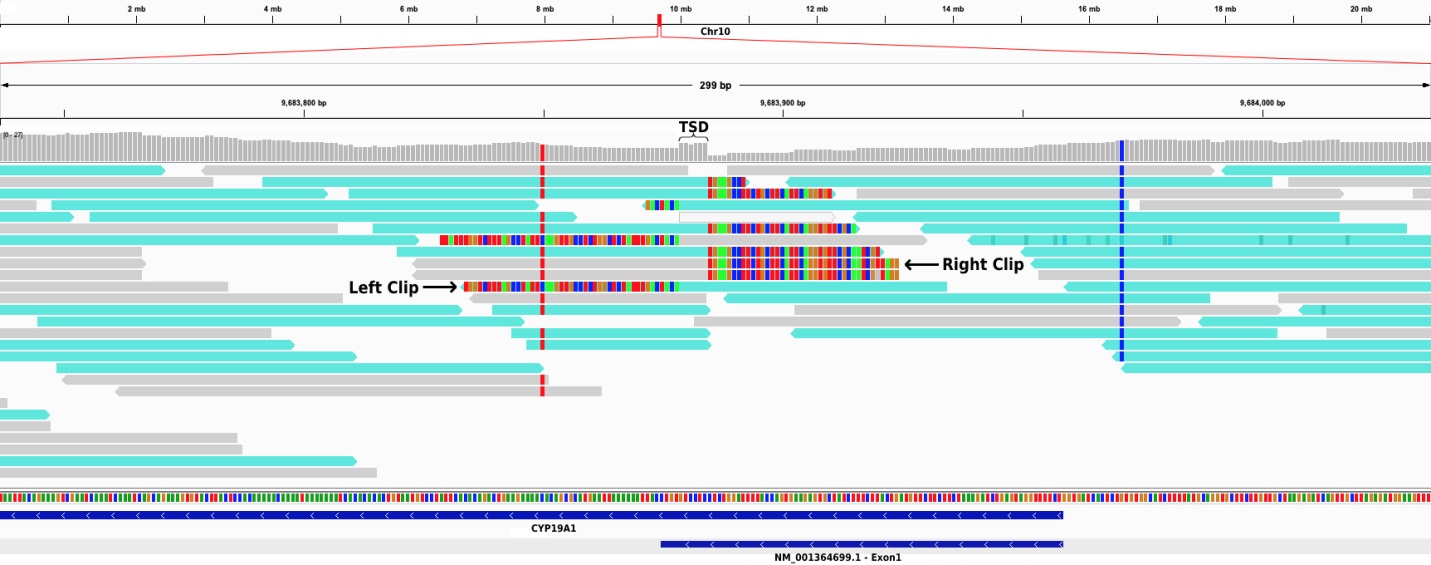

Supplement: Supplementary file 5 — Figure S1. Aligned reads showing the insertion site of Hf_ERV on chicken chromosome 10. Aligned reads are shown in gray; multicolored “rainbow” reads are soft-clipped. Left clip and right clip sequences match the 5′ and 3′ of the LTR sequence, respectively. Discordant reads are in cyan which are well aligned in this figure (Chr10), while their mate pairs are aligned to Chr1, either to LOC770705 or LOC107052718, both retrovirus related genes, because there is no retrovirus sequence at this target site on chromosome 10 in the reference genome. Altogether, this alignment suggests a retrovirus insertion located between the left and right clips, which contains the 6 bp target site duplication (TSD) sequence. Reads are obtained from BioSample Accession SAMEA104432213 (Pool of Silver Sebright Bantam) and aligned to GalGal6. (DOCX 214 kb) [file 13100_2019_181_MOESM5_ESM.docx]

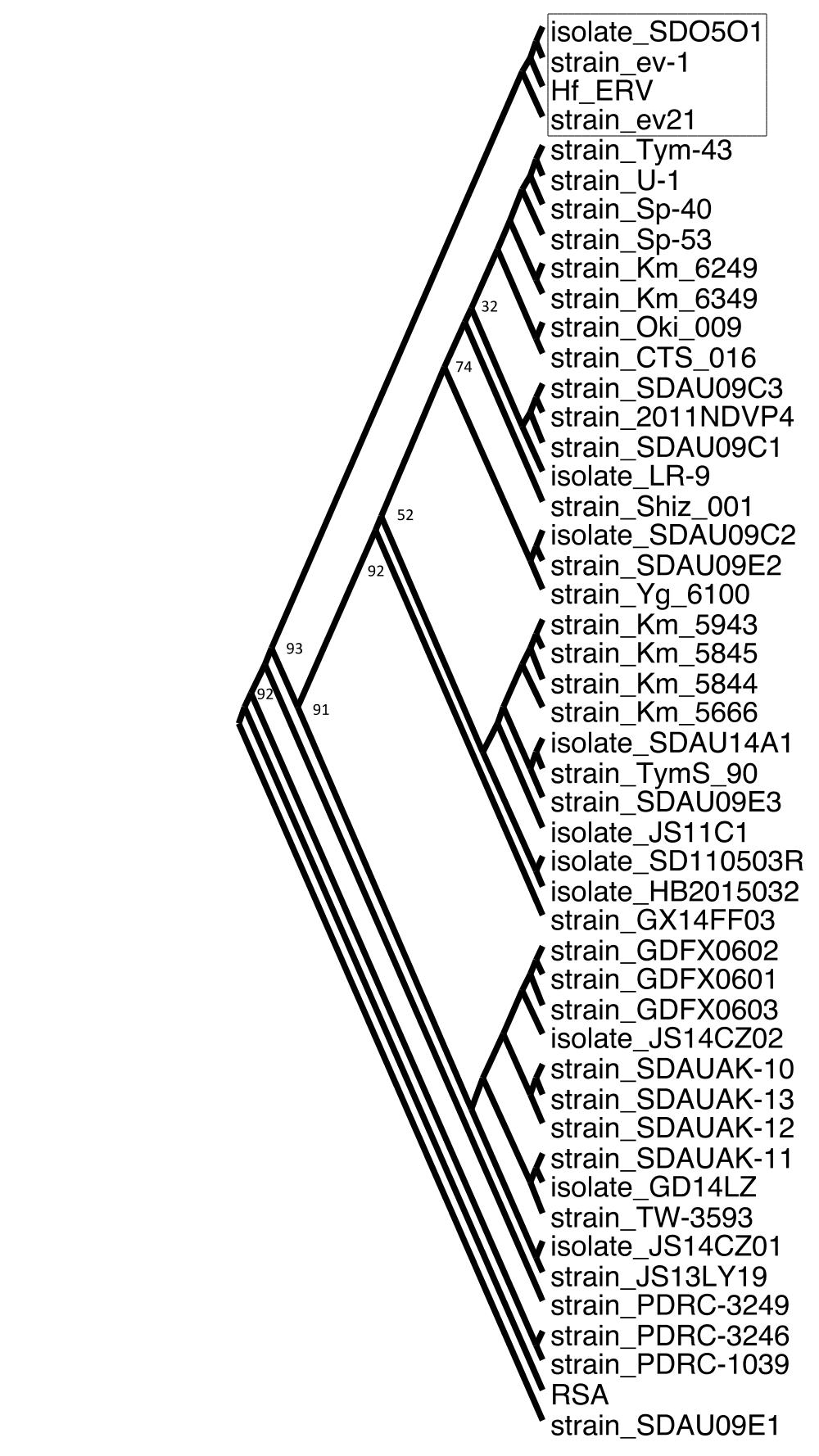

Supplement: Supplementary file 6 — Figure S2. Maximum likelihood phylogeny for the endogenous retrovirus associated with henny feathering (Hf_ERV). Hf_ERV and the 50 other retrovirus sequences with above 90% sequence identity to Hf_ERV. Bootstrap values are reported as percentages. Grey box indicates the clade containing Hf_ERV, ev-1 and ev21. (DOCX 475 kb) [file 13100_2019_181_MOESM6_ESM.docx]

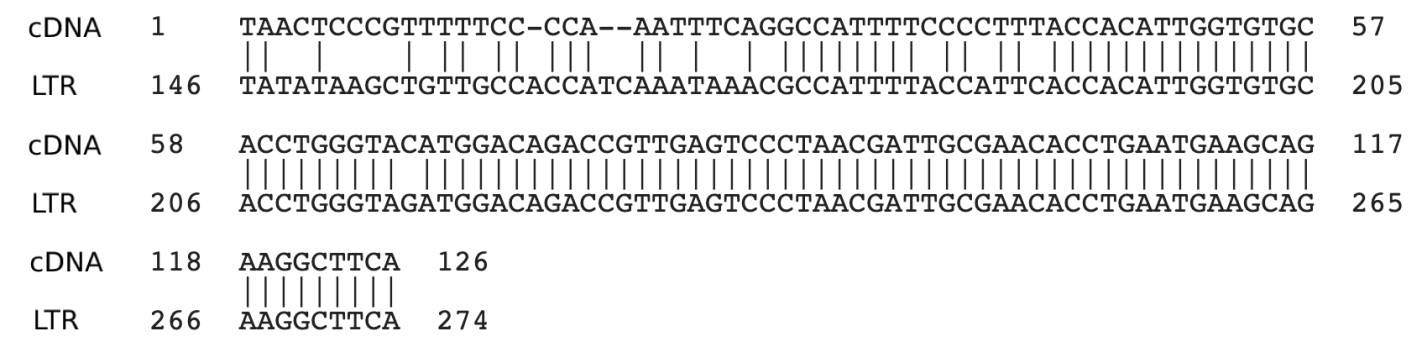

Supplement: Supplementary file 7 — Figure S3. Comparison of sequences reported in this and previous studies. Alignment between a previously reported partial cDNA sequence of CYP19A1 in Sebright chickens [21] and the retrovirus insertion detected in the present study (LTR). The base pair positions of the LTR sequence correspond to the complete LTR (5′ to 3′), which is 274 bp in length. (DOCX 140 kb) [file 13100_2019_181_MOESM7_ESM.docx]
